# Supplementary material for: Splice-Junction-Based Mapping of Alternative Isoforms in the Human Proteome
Source: Cell Rep. Author manuscript; Available in PMC 2020 Jan 15. (PMC6961840; doi:10.1016/j.celrep.2019.11.026)
Supplement: 2 [file NIHMS1546469-supplement-2.zip › DF1/PXD009737/TestisTrypsin/TestisTrypsin_20_PSME4_TEHLGLNWFPK.pdf]

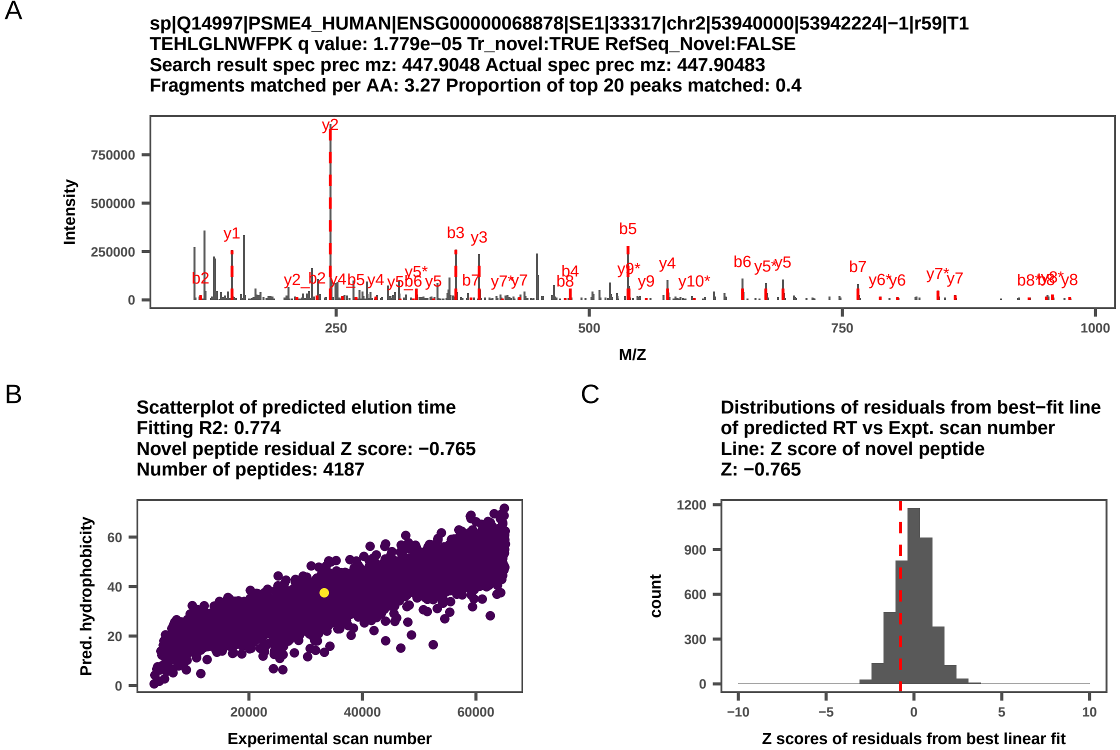
A
sp|Q14997|PSME4_HUMAN|ENSG00000068878|SE1|33317|chr2|53940000|53942224|−1|r59|T1
TEHLGLNWFPK q value: 1.779e−05 Tr_novel:TRUE RefSeq_Novel:FALSE
Search result spec prec mz: 447.9048 Actual spec prec mz: 447.90483
Fragments matched per AA: 3.27 Proportion of top 20 peaks matched: 0.4
Intensity
M/Z
B
Scatterplot of predicted elution time
Fitting R2: 0.774
Novel peptide residual Z score: −0.765
Number of peptides: 4187
Pred. hydrophobicity
Experimental scan number
C
Distributions of residuals from best−fit line
of predicted RT vs Expt. scan number
Line: Z score of novel peptide
Z: −0.765
count
Z scores of residuals from best linear fit
